# Pinostilbene as a Potential Cytotoxic Agent in Cancer Cell Lines: Improvement of Solubility and Stability by Cyclodextrin Encapsulation

**DOI:** 10.3390/pharmaceutics17091219

**Published:** 2025-09-19

**Authors:** Irene Conesa, Silvia Navarro-Orcajada, Francisco José Vidal-Sánchez, Elena Torralba-Antón, Marta Carrión-Espinosa, Adrián Matencio, José Manuel López-Nicolás

**Affiliations:** Departamento de Bioquímica y Biología Molecular-A, Facultad de Biología, Regional Campus of International Excellence “Campus Mare Nostrum”, Universidad de Murcia, E-30100 Murcia, Spain; irene.conesav@um.es (I.C.); silvia.navarro6@um.es (S.N.-O.); fjose.vidal@um.es (F.J.V.-S.);

**Keywords:** pinostilbene, stilbene, cancer, encapsulation, solubility, stability

## Abstract

**Background/Objectives:** Pinostilbene is a naturally occurring methoxylated stilbene with many beneficial health properties, including antioxidant, antimicrobial and neuroprotective activities. This stilbene has also been shown to possess anticancer or cytotoxic activity in some cancers. As in the case of other stilbenes, pinostilbene is very labile, degrades rapidly under stress conditions and is poorly water-soluble, which poses a drawback to its use as a drug. This work aims to provide further insights into its cytotoxicity activity in a colon cancer cell line and to overcome its physicochemical limitations by encapsulating the molecule in cyclodextrins. **Methods:** The anticancer activity was evaluated in vitro in Caco-2 colorectal cells using the neutral red assay. Subsequently, a screening of cyclodextrins was carried out to determine the one with the highest encapsulation constant, as well as the encapsulation stoichiometry, using fluorescence spectroscopy and molecular docking predictions. The formation of the inclusion complexes was checked by differential scanning calorimetry and scanning electron microscopy. The protective effect of cyclodextrins on pinostilbene release was monitored through spectrophotometric measurements over time. **Results:** Pinostilbene showed in vitro cytotoxicity activity in Caco-2 colorectal cells by the neutral red assay. This study revealed that the cyclodextrin with the highest encapsulation constant was the hydroxypropyl-β-cyclodextrin (K_F_ = 10,074.45 ± 503.72 M^−1^), and the encapsulation stoichiometry was 1:1. DSC and SEM assays confirmed the formation of these inclusion complexes. Cyclodextrins were able to satisfactorily reduce pinostilbene degradation from 31% to less than 15% after 3 months, as well as increase its water solubility up to 10 times and enhance its release as a function of the pH of the medium. **Conclusions:** Pinostilbene is a promising drug candidate with strong in vitro antiproliferative activity. Many of its physicochemical limitations can be overcome with cyclodextrins, which opens the door to its future use in the pharmaceutical and food industries.

## 1. Introduction

In recent years, there has been increasing interest among consumers in having a healthier lifestyle. As a response to this global demand, research on bioactive compounds and their beneficial properties is rising, to incorporate these molecules into cosmetics and functional foods or even to develop novel drug therapies. There are innumerable sources of bioactive compounds in nature; however, plant secondary metabolites are, without a doubt, the richest and most diverse source of interesting compounds with healthy properties. Plant secondary metabolites are divided in three main families: alkaloids, terpenes and phenolic compounds [1]. While there are numerous molecules with bioactive properties in all three families, phenolic compounds have a vast variety of molecular diversity and beneficial activities: antioxidant, anticancer, neuroprotective and anti-inflammation, among many other properties [2].

Regarding phenolic compounds, stilbenes are a group of polyphenols that share the diphenylethylene backbone. There are numerous interesting members with different substituents like hydroxyl or methoxyl groups, with resveratrol (3,4′,5-trihydroxystilbene) being the most widely studied. Resveratrol has been proven to have a strong antioxidant and anticancer capacity, among a long list of bioactivities described in vitro and in vivo. However, not only does resveratrol present these bioactive properties, it also the most bioactive member of the stilbene family, though other stilbenes like oxyresveratrol, gnetol and isorhapontigenin are increasingly being studied and are being reported to have—in some cases—stronger bioactive properties than resveratrol itself [3].

**Figure 1:**
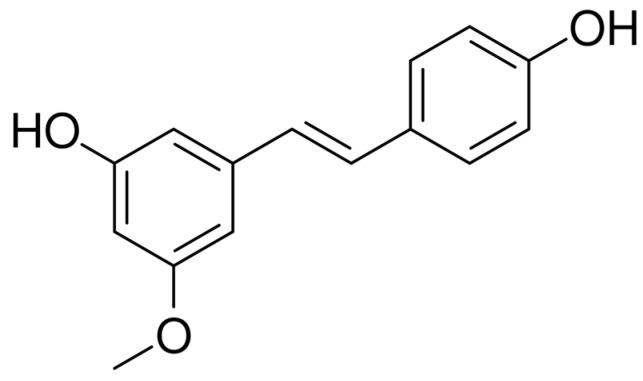
Molecular structure of pinostilbene.

One of these stilbenes with little information available is pinostilbene (*trans*-3,4′-dihydroxy-5-methoxystilbene, (Figure 1), which is a methoxylated stilbene naturally occurring in *Pinus sibirica* [4]. However, it is usually synthesized from resveratrol in cultures modified with O-methyltransferases enzymes such as those of *Vitis vinifera* [5], *Vitis riparia* or *Sorghum bicolor* [6], or directly in plant cultures of *Phytolacca americana* [7]. In mice, pinostilbene has been found to be the main metabolite of pterostilbene [8]. The interest in this stilbene lies in its bioactivity as an antimicrobial [9], anti-inflammation [10], neuroprotective [11] and anti-obesity [12] compound. Pinostilbene has also shown anticancer or cytotoxic activity by modulating proteins involved in cell proliferation, apoptosis [8] or migration in different types of cancer, such as oral carcinoma [13], prostate cancer [4] and multiple myeloma [14], among others. Furthermore, in colonic fermentation assays, it proved to be more stable as it was not metabolized to new products, unlike other stilbenes such as resveratrol, oxyresveratrol and piceatannol [15]. Like other methoxylated stilbenes, its structure may play a beneficial role in the bioavailability of the molecule, making it easier to cross the cell membranes and to perform its biological activity within the cells, yet limiting its aqueous solubility.

To maintain the beneficial properties of pinostilbene and reduce the drawbacks that limit its use, such as low stability and aqueous solubility, the molecular encapsulation of this stilbene is proposed [16]. Encapsulation occurs when a stilbene is embedded in a matrix, facilitating its release and protecting the molecule from degradation by external factors. It is not a fixed process; a dynamic equilibrium is established between the formation of inclusion complexes and the free molecules. This process can be carried out, among others, using cyclodextrins (CDs) [17].

**Figure 2:**
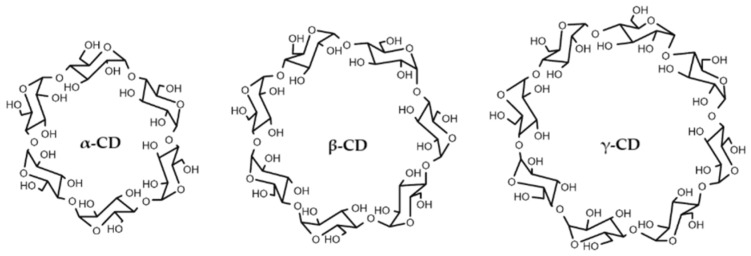
Molecular structure of natural cyclodextrins.

CDs are truncated cone-shaped oligosaccharides consisting of glucose molecules linked by an α (1→4) bond. CD rings have a polar outer surface and a non-polar internal cavity, which allows them to accommodate poorly water-soluble molecules, such as stilbenes, within forming inclusion complexes. The most common CDs are the natural types, α-CD, β-CD and γ-CD (Figure 2), which consist of six, seven and eight glucose units, respectively, and are obtained from the degradation of starch by the enzyme cyclodextrin glucosyltransferase [17]. Similarly, we also find CDs modified from natural ones, which in some cases may have bioactivities of their own, such as the use of hydroxypropyl-β-cyclodextrin for the treatment of Niemann–Pick type C disease [18] or gout [19]. Additionally, they are well known for increasing the solubility and stability of drugs, bioactive compounds [3,20] and other stilbenes, such as gnetol [21], rhapontigenin [22], isorapontigenin [23] and oxyresveratrol [24], among others.

In this work, the cytotoxic activity of pinostilbene and its analogue resveratrol was studied on a human colorectal cancer cell line (Caco-2). The encapsulation of pinostilbene in different CDs was also studied, determining the complex formation constant (K_F_) for each of them. Furthermore, the efficacy of the CDs in increasing the solubility and stability of this stilbene in aqueous solutions was also evaluated.

## 2. Materials and Methods

### 2.1. Materials

Natural CDs (α-CD, β-CD and γ-CD) were purchased from Sigma Aldrich (Madrid, Spain). Methyl-β-cyclodextrin (M-β-CD, DS = 5.4) and 2-hydroxypropyl-β-cyclodextrin (HP-β-CD, DS = 5) were purchased from Carbosynth (Berkshire, UK). Pinostilbene (>97% purity) and resveratrol (>99% purity) were purchased from TCI (Haven, Belgium). Sodium phosphate monobasic dihydrate (>99% purity) was purchased from Merck-Sigma Aldrich (Madrid, Spain). All solvents were of HPLC grade and were purchased from Fisher Scientific (Alcobendas, Spain).

### 2.2. Methods

#### 2.2.1. Cell Line and Culture Conditions

Human colorectal cancer cell line Caco-2 was obtained from American Type Culture Collection (ATCC, Rockville, MD, USA) and was cultured in complete EMEM growth medium with 10% (*v*/*v*) foetal bovine serum and 0.1 mg/mL penicillin/streptomycin. Cells were grown according to ATCC guidelines [25] in an incubator (Thermo Scientific model BBK 6220, Waltham, MA, USA) at 37 °C, 5% CO_2_ and 85% relative humidity.

#### 2.2.2. Cytotoxicity Test

Caco-2 cells (passage 34–37) were seeded in 96-well plates at 10,000 cells per cm^2^, filling the peripheral wells with sterile water to avoid evaporation effects. Cell concentration was determined with trypan blue using a TC10™ automated cell counter (Bio-Rad, Madrid, Spain). After incubation for 48 h, the culture medium was replaced with fresh medium supplemented with 25, 50 and 100 µM of resveratrol or pinostilbene. All tested compounds were solubilized in dimethyl sulfoxide (DMSO 0.33% in the culture medium) and filter sterilized (0.2 µm Merk Milipore, Burlington, MA, USA) before addition to the culture medium. Control cells (untreated) containing 0.33% DMSO were run in parallel and subjected to the same changes in medium. Each treatment was performed six times.

Cell proliferation was measured after 48 h of incubation with the treatments by neutral red assay [26]. Briefly, a neutral red stock solution was prepared at 4 mg/mL in PBS and stored at room temperature in darkness. Treated cells were washed with PBS and incubated for 2 h with a working solution of neutral red (dilution 1:100 of the stock solution in complete growth medium). The cells were then washed again with PBS and air dried, and an unstaining solution (consisting of a mixture of ethanol, water and acetic acid (1:1:0.02)) was added. After 10 min of gentle oscillation, the absorbance was read at 540 nm on a FLUOstar Omega plate reader (BMG Labtech, Ortenberg, Germany). Background absorbance at 690 nm was subtracted from the measurement, as well as the absorbance of a blank with no cells. The relative cell viability of each treatment was determined by comparison to the control.

To compare the IC_50_ of pinostilbene and resveratrol calculated in this work with other stilbenes, data from a previous rhapontigenin [22] and isorhapontigenin [23] assay carried out under the same conditions were requested.

#### 2.2.3. Determination of Stoichiometry and Encapsulation Constants

The Benesi–Hildebrand method [27] proposes two possible mathematical models depending on the stoichiometry of the complexes: a 1:1 model in which a single CD molecule binds to each molecule of the bioactive compound, and a 1:2 model in which two CD molecules bind to each molecule of the bioactive compound.

(1) Pinostilbene+CD   ⇌  Pinostilbene−CD

(2) Pinostilbene+2CD   ⇌  CD−Pinostilbene−CD

Considering both models, the encapsulation or formation constant (*K_F_*) of pinostilbene was calculated using the following expression:

(3) $$K_{F}=\frac{\left[Inclusion\;complex\right]}{\left[Pinostilbene\right]\cdot {\left[CD\right]}^{x}}$$

where [*Inclusion complex*], [*Pinostilbene*] and [*CD*] are the concentrations at equilibrium of the inclusion complex, ligand and CD, respectively, and *x* is the stoichiometric coefficient (*x* = 1 for the 1:1 model, *x* = 2 for the 1:2 model).

The encapsulation constant is a parameter of special interest for characterizing the interaction between CDs and ligands. The higher its value, the greater the interaction between molecules, and therefore, the more stable the inclusion complex, which allows for screening CDs for the same bioactive compound.

Experimentally, the encapsulation constant was obtained with relative fluorescence measurements at increasing CD concentrations using the following equation:

(4) $$\frac{1}{F-F_{0}}=\frac{1}{\left(F_{\infty }-F_{0}\right)\cdot K_{F}\cdot [{CD]}^{x}}+\frac{1}{F_{\infty }-F_{0}}$$

where *F*_0_ is the basal fluorescence intensity of pinostilbene in the absence of CDs, *F_∞_* is the fluorescence intensity when all the molecules of pinostilbene are encapsulated in CDs, *F* is the observed fluorescence intensity at each CD concentration, *K_F_* is the encapsulation constant, [*CD*] is the CD concentration and *x* is the stoichiometric coefficient.

#### 2.2.4. Molecular Docking

Pinostilbene structure was obtained from the PubChem database [28] (NCBI, USA) (PubChem CIB 5473050). The molecular structures of natural CDs α-CD and β-CD were extracted from their crystal structures obtained in the Protein Data Bank (PDB ID: 2XFY and 1Z0N, respectively), while γ-CD was obtained from the London South Bank University website [29]. Modified CDs HP-β-CD and M-β-CD were constructed by adding hydroxypropyl or methyl groups to the β-CD structure. The topology of the modified CDs was obtained using PRODRG [30] with default parameters. For other molecules used, their default topology was maintained. AutoDock tools (version 1.5.6) with default parameters and charges was used to generate input files for molecular docking. Molecular docking was performed using AutoDock Vina (version 1.1.2) [31] with default parameters, setting a seed of 5000 and considering flexible atoms in the structure of CDs. Only the best pose was analyzed.

The final representations were prepared using PyMOL (Molecular Graphics System, version 1.3, Schrödinger, LLC, New York, NY, USA). To facilitate visualization, the ligand structure was displayed as sticks, while the receptor structure was displayed as lines, with hydrogens hidden. Flexible carbon atoms of CDs were colored orange, while non-flexible carbon atoms of CDs were in blue. Carbon atoms of the ligand were colored green, while aromatic rings were automatically shown in white. The visualization of hydrogen bonds was obtained with default parameters and were displayed as discontinuous yellow lines.

#### 2.2.5. Fluorimetric Studies and Determination of pH Influence

The pinostilbene concentration was fixed to 25 μM (from an ethanolic solution at 4.13 mM), and the CDs were set from 0 to 10 mM in several buffers, as follows. The pH was fixed in all assays using buffers previously prepared: 0.1 M sodium phosphate buffer pH 5; 0.1 M sodium phosphate buffer pH 7; 0.1 M sodium borate buffer pH 8; 0.1 M sodium borate buffer pH 9; and 0.1 M sodium borate buffer pH 10. All samples were vortexed for 30 s and incubated for 30 min in the dark before measuring their fluorescence intensity. Measurements were performed on a Kontron SFM-25 spectrofluorometer (Zurich, Switzerland) equipped with thermostatically controlled cells at 25 °C, a xenon light source and 2 mm quartz cells. The excitation and emission bandwidths were set to 2 nm. The wavelengths of fluorescence maximum excitation and fluorescence maximum emission used were previously determined using a Shimadzu RF-6000 spectrofluorometer (Kyoto, Japan) equipped with thermostatically controlled cells, setting the excitation and emission bandwidths to 5 nm.

#### 2.2.6. Determination of Aqueous Solubility

To determine the water solubility of the bioactive compound, a published protocol was used with few modifications [22]: saturated aqueous solutions of pinostilbene were prepared at a theoretical concentration of 1 mg/mL in water, although only a small proportion was soluble. In the case of assays with CDs, these were added during preparation at concentrations between 1 and 10 mM. Samples were agitated using a vortex and incubated at 25 °C in the dark for 10 min. Subsequently, they were centrifuged at 15,600× *g* for 1 min (Spectrafuge 24D Labnet, Edison, NJ, USA) to remove the excess of pinostilbene (free drug), and the supernatant fraction containing the dissolved stilbenes was separated. The supernatants were diluted 1:100 in ethanol, and their absorbance at the previously established maximum absorbance wavelength was measured using a Jasco V-650 spectrophotometer (Jasco, Madrid, Spain) with Thorlabs CV10Q1400 cuvettes. A solution with ethanol and water at the same final concentration as the samples was used as a blank. The concentration of soluble pinostilbene was determined by Lambert–Beer’s law, previously establishing the molar attenuation coefficient (ε) of pinostilbene by measuring the absorbance between 200 and 600 nm of increasing concentrations of stilbene in an ethanolic solution.

#### 2.2.7. Determination of Stability in Aqueous Solution

Solutions containing pinostilbene at 25 µM in 0.1 M phosphate buffer pH 7 were prepared and stored at room temperature in the dark for a maximum of 3 months (laboratory heated to a constant 25 °C). In the case of assays with CDs, these were added during preparation at concentrations between 1 and 10 mM. The remaining percentage of pinostilbene was monitored every two weeks by measuring absorbance at the maximum absorbance wavelength previously determined using a Jasco V-630 spectrophotometer (Madrid, Spain) with Thorlabs CV10Q1400 cuvettes. A solution with buffer and ethanol at the same final concentration as the samples was used as a blank. Results were expressed as the percentage of remaining pinostilbene using the following formula:

(5) $$Remaining\;pinostilbene\;\left(\%\right)=\frac{{Abs}_{i}}{{Abs}_{0}}\cdot 100$$

where *Abs_i_* is the absorbance at each time *i*, and *Abs*_0_ is the absorbance at time 0.

#### 2.2.8. Pinostilbene Complexation in HP-β-CD and Characterization

The complex was prepared using a kneading approach to prevent eventual degradation using an existing protocol with little modification [32]. Briefly, 10 mg of pinostilbene were added to a mortar with 90 mg of HP-β-CD to obtain a 10% loading. Then, 30 µL of 50/50 deionized H_2_O/ethanol was added to the sample and manually mixed for 60 min. The physical mixture was prepared by mixing for 5 min at the same proportion, but without adding any solvent. The powder was collected and dried in the air. The complex was confirmed using a Jasco V-650 spectrophotometer (Madrid, Spain) with Thorlabs CV10Q1400 cuvettes. These complexes were used for structural characterization and solubility testing. In their formation, excess CD is used to ensure that all the pinostilbene used is complexed.

A differential scanning calorimetry (DSC) assay was conducted using an SDT instrument (model 2960, TA Instruments, New Castle, DE, USA), increasing the temperature from 40 to 200 °C at a 10 °C/min gradient. All DSC analyses were performed under pure nitrogen with 5 mg of sample.

The surface morphology of the samples was examined by scanning electron microscopy (SEM) using a FESEM (field emission scanning electron microscope) (ApreoS, Thermofisher, Waltham, MA, USA) at high vacuum at 1 kV. The samples were prepared on an aluminum support and covered with carbon to improve their conductivity.

#### 2.2.9. In Vitro Release Studies

The release of pinostilbene from the complex was studied by a membrane diffusion method [33,34]. Dialysis membranes (cut-off 1 kDa) were submerged in different stirred flasks at 37 °C with 50 mL of PBS (10 mM sodium phosphate buffer + 140 mM NaCl) at pH 7.4 or 6. The donor phase comprised different nanosuspensions consisting of a suspension of 1 mg of pure pinostilbene or a pinostilbene-loaded suspension. The receptor phase also contained the same medium. Different samples were taken, and the receptor phase was diluted with the same amount of fresh buffer. The samples were analyzed through UV spectroscopy using the peak at 307 nm in the Jasco V-650 spectrophotometer (Madrid, Spain).

To evaluate the drug release mechanism, release patterns were analyzed in vitro using zero-order kinetic models. In zero-order kinetics, the rate of drug release is constant, regardless of the drug concentration [35] (Equation (6)).

(6) $$C=\frac{C_{t}}{1+k[CD]}$$

*C* is the free concentration of the drug over time, *C_t_* is the total concentration, *k* is the zero-order rate constant and [CD] is the CD concentration.

#### 2.2.10. Data Analysis

All experiments were performed in triplicate. Regressions were performed with Sigma-Plot (version 10.0.0.54). A *t*-test was performed using RStudio (version 0.99.878) with a significance of *p* < 0.05.

## 3. Results and Discussion

### 3.1. Inhibition of Caco-2 Proliferation After Pinostilbene Treatment

**Figure 3:**
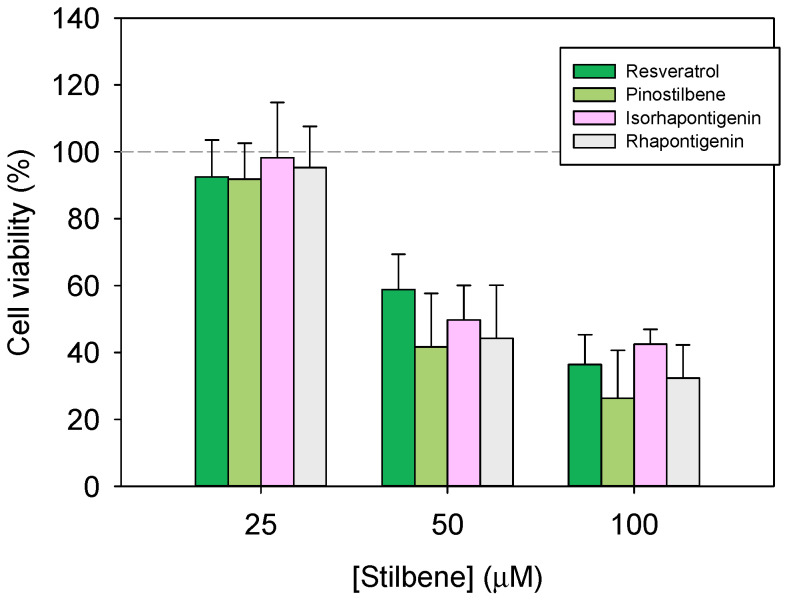
Cytotoxicity of pinostilbene, resveratrol, isorhapontigenin (data obtained from [23]) and rhapontigenin (data obtained from [22]) on human colorectal cancer cell line (Caco-2) after 48 h of treatment.

After 48 h of incubation with pinostilbene and its analogue, resveratrol, cell viability decreased in a dose-dependent manner (Figure 3). The lowest concentration of both stilbenes showed no significant effect on the inhibition of cell proliferation; however, concentrations above 25 µM decreased cell viability notably. At 100 µM of pinostilbene, cell viability was 26%, whilst for the same concentration of resveratrol, it was 36% (Figure 3). According to previous studies conducted on other colon cancer cell lines (HT29 and HCT116), pinostilbene may exert its action by modulating the expression of key proteins involved in cell proliferation and apoptosis [8].

In order to compare the results with the previous literature, IC_50_s were estimated from the above data. Resveratrol showed an IC_50_ of 76.20 ± 10.8 µM, while pinostilbene showed an IC_50_ of 62.53 ± 13.4 µM. Data requested from the same studies with isorhapontigenin [23] and rhapontigenin [22] also allowed for the calculation of their IC_50_s, which are located at 78.95 ± 10.5 µM and 67.95 ± 12.7 µM, respectively. When compared, all these stilbenes have similar cytotoxicity. This stilbene order, according to their cytotoxicity, correlates with that obtained in other studies determining the IC50 of resveratrol and pinostilbene in the colon cancer lines Caco-2 and HT-29 [36]. In addition, other studies have shown that methoxylated analogues have more potent anti-mitogenic effects on the Caco-2 cell line than their hydroxylated analogues, but without effects on oxidative stress and the arachidonic acid cascade [37].

### 3.2. Physicochemical Study of the Inclusion Complexes with Pinostilbene

**Figure 4:**
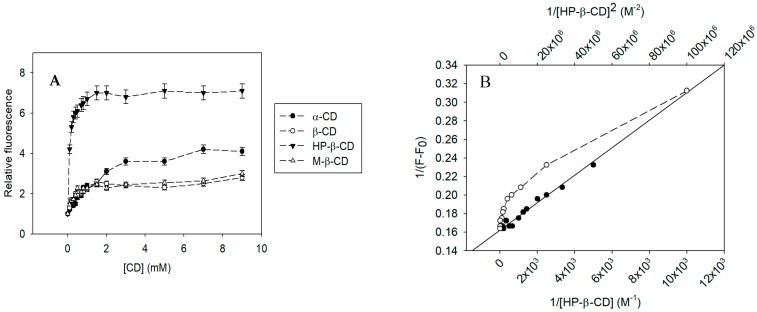
Complexation curve of pinostilbene in CD: (**A**) Effect of increasing (●) α-CD, (○) β-CD, (▼) HP-β-CD or (△) M-β-CD concentrations on pinostilbene fluorescence. (**B**) Stoichiometric fitting of pinostilbene inclusion complexes with HP-β-CD to a (●) 1:1 and (○) 1:2 model by the Benesi–Hildebrand method (25 °C, pH 7).

Fluorimetric measurements were carried out using the excitation and emission maximums of pinostilbene: 290 nm and 397 nm, respectively. The molecular encapsulation of pinostilbene in CDs (Figure 4A) showed a complexation curve similar to that previously observed with other stilbenes. Except for γ-CD, all CDs increased the basal fluorescence of pinostilbene as a function of the concentration used. Through the Benesi–Hildebrand fit (Figure 4B), it was possible to determine that the complexation followed a 1:1 stoichiometry, in which one CD molecule interacts with one molecule of pinostilbene. This type of interaction is in good accordance with that previously observed in the molecular encapsulation of other stilbenes, such as isorhapontigenin [23], rhapontigenin [22], gnetol [21], resveratrol [38], pterostilbene [39], piceatannol [40] and oxyresveratrol [41].

The encapsulation constants of the pinostilbene complexes with the different CDs were determined using Equation (4) adjusted for the 1:1 model (Table 1). The results show that HP-β-CD was the most suitable CD for the molecular encapsulation of pinostilbene, given its higher encapsulation constant (K_F_ = 10,074.45 ± 503.72 M^−1^), followed by M-β-CD (K_F_ = 4870.24 ± 243.51 M^−1^), β-CD (K_F_ = 3503.62 ± 175.18 M^−1^), α-CD (K_F_ = 633.62 ± 31.68 M^−1^) and finally, γ-CD, for which a linear fit to determine the encapsulation constant could not be obtained.

The values are in agreement with that shown in our previous studies with different stilbenes [21,22,23,40], where it was also observed that the most stable inclusion complexes between these compounds and CDs were obtained with β-CD and its semi-synthetic derivatives, with HP-β-CD being especially notable at similar values.

### 3.3. Molecular Docking of Pinostilbene in CDs

The computational results showed a good correlation with the experimental results regarding the order of preference of CDs for the encapsulation of pinostilbene. The highest score was given by the complex with HP-β-CD (score = −10.1), followed by the complexes with M-β-CD (score = −9.2), β-CD (score = −9.1), α-CD (score = −6.8) and lastly, γ-CD (score = −6.2).

**Figure 5:**
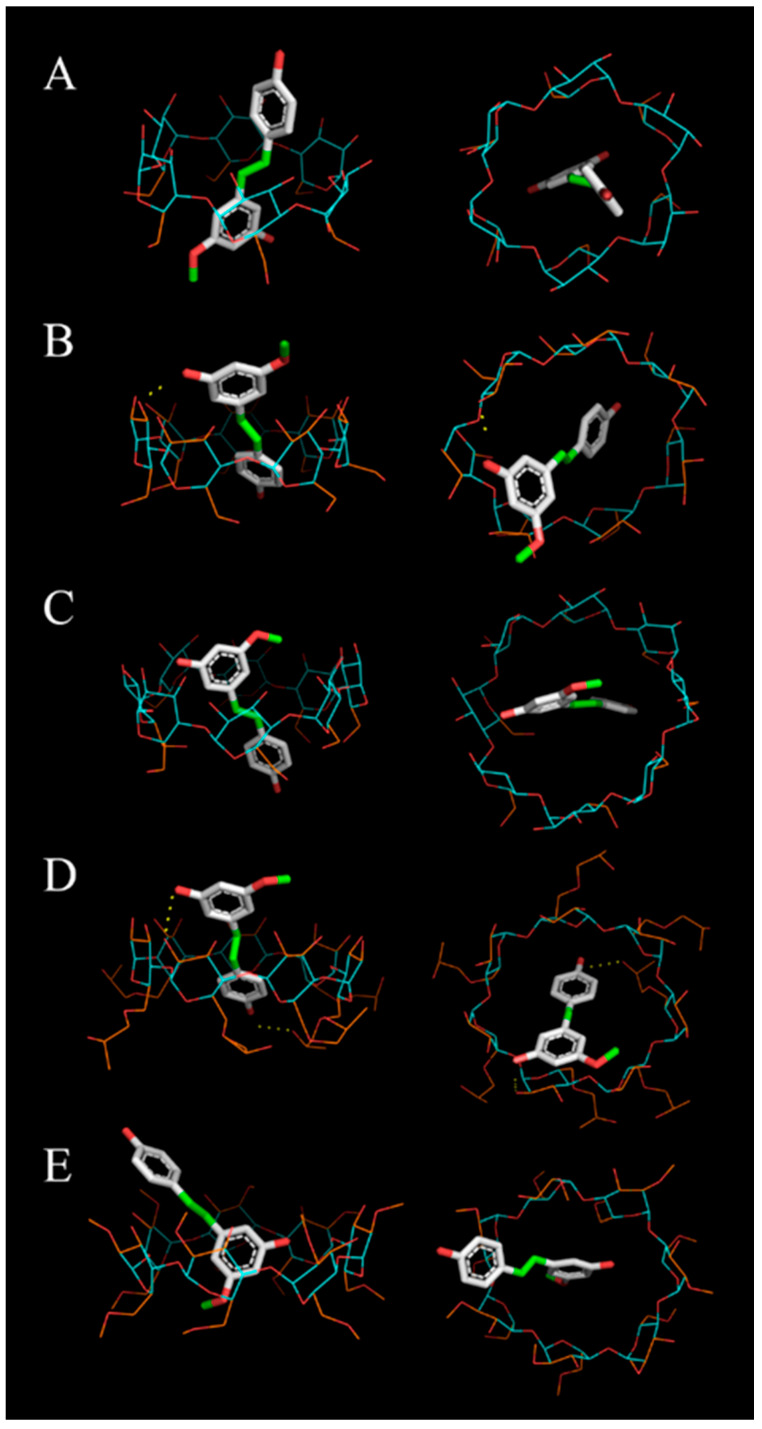
Three-dimensional molecular docking of pinostilbene in (**A**) α-CD, (**B**) β-CD, (**C**) γ-CD, (**D**) HP-β-CD and (**E**) M-β-CD. The flexible atoms of CD are displayed in orange, while the non-flexible atoms are displayed in blue. Possible hydrogen bonds are represented as yellow dotted lines (shown in (**B**,**D**)).

In the most likely configurations shown in Figure 5, hydrogen bonds were observed between pinostilbene and two of the best-scoring CDs: β-CD and HP-β-CD (Figure 5B and Figure 5D, respectively). It is possible that in the complex with M-β-CD (Figure 5E)—where no hydrogen bonds are observed but a similar score to β-CD was obtained—other types of weak interactions, such as hydrophobic or Van der Waals interactions, stabilize the binary complexes. The lower-affinity molecular dockings, i.e., with α-CD and γ-CD (Figure 5A,C), revealed that the pinostilbene was in a more centered position, leaving free spaces in the CD cavity. This suggests that these complexes may have fewer interactions that stabilize ligand–receptor coupling.

### 3.4. Influence of pH on Pinostilbene Encapsulation

**Figure 6:**
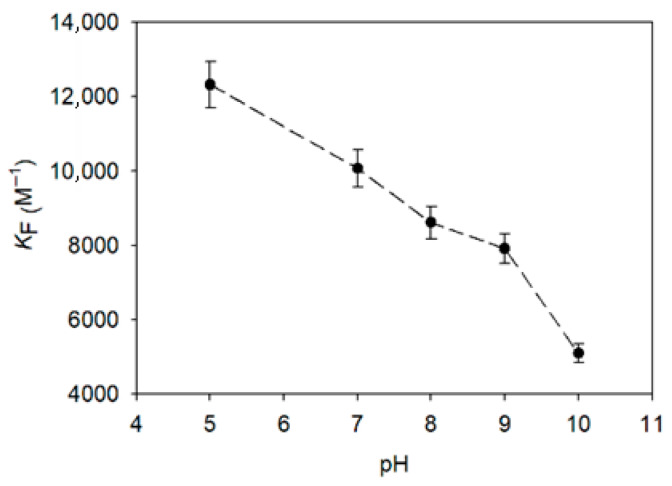
Effect of pH on the encapsulation constants of the HP-β-CD–pinostilbene inclusion complexes.

The encapsulation constants of the pinostilbene complexes with the best CD obtained by the Benesi–Hildebrand method and the molecular docking, HP-β-CD, were inversely related to the pH of the medium. The higher the acidity in the medium, the higher the encapsulation constants given by the inclusion complexes, reaching up to K_F_ = 12,322.75 ± 616.14 M^−1^ at pH 5 (Figure 6). On the contrary, when the pH of the medium became more basic, these values dropped, reaching the minimum value of K_F_ = 5105.60 ± 255.28 M^−1^ at pH 10. From pH 5 to pH 9, the decrease in the K_F_ value was constant (R^2^ > 0.99), but from pH 9 onwards, it intensified, doubling the negative slope. As described above [21,40], this intensified decrease has been associated with the pH region where the pK_a_ of the stilbene is located, although our method was not sensitive enough to discern between the pKas separately.

### 3.5. Solubility of Free and Encapsulated Pinostilbene

**Figure 7:**
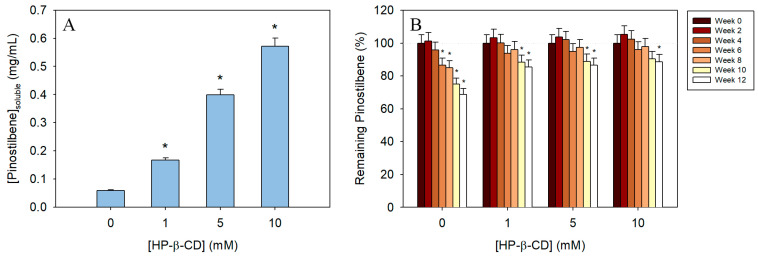
(**A**) Aqueous solubility of pinostilbene in the absence and presence of different concentrations of HP-β-CD. (**B**) Stability of free and encapsulated pinostilbene in HP-β-CD after storage at room temperature (25 °C). * Significance *p*-value < 0.05.

Solubility studies of free and complexed pinostilbene highlighted that molecular encapsulation increases the aqueous solubility of this stilbene significantly (Figure 7A). While in non-complexed pinostilbene solubility in water was 0.06 mg/mL (ε = 32,060 M^−1^cm^−1^), the addition of 1 mM HP-β-CD tripled this value. The use of higher concentrations of CD also made significant differences, achieving up to 7- and 10-times more soluble stilbene with 5 mM and 10 mM HP-β-CD, respectively (Figure 7A).

The solubility of pinostilbene was closer to that of pterostilbene (0.021 mg/mL [42]) and resveratrol (0.03 mg/mL [43]), with which it shared the positions of its radicals, but was lower than that of other stilbenes with substitutions in other groups, such as oxyresveratrol (0.75 mg/mL [44]), piceatannol (0.50 mg/mL [43]), gnetol (0.31 mg/mL [21]), isorhapontigenin (0.32 mg/mL [23]) and rhapontigenin (0.11 mg/mL [22]).

### 3.6. Time-Course Evaluation of the Stability of the Inclusion Complexes

On the other hand, the room-temperature stability of pinostilbene also improved after molecular encapsulation (Figure 7B). The degradation of pinostilbene after storage in free form was milder compared to that observed for other stilbenes [22,23]. From week 6 onwards, this decrease in pinostilbene concentration was already significant compared to the initial concentration. After 12 weeks of storage, the solutions without CDs had lost as much as 31% of the initial pinostilbene, whereas in the presence of HP-β-CD, this loss did not exceed 15% of the initial pinostilbene concentration, with more than 88% of the initial amount preserved using a concentration of CD of 10 mM.

### 3.7. DSC Analysis

By measuring heat flow associated with thermal transitions, DSC provides critical insights into changes in melting points, enthalpy variations and the thermal stability of the components and their complexes. The formation of an inclusion complex often results in the disappearance or a significant shift of the guest thermal events, indicating successful encapsulation [24,33].

**Figure 8:**
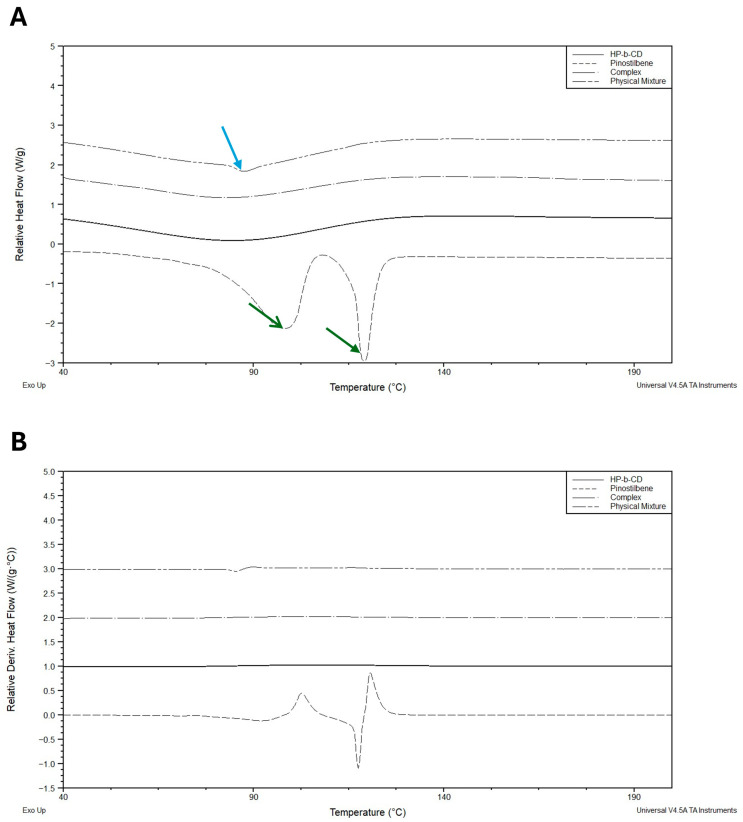
Comparison of differential scanning calorimetry (DSC) results of the different starting materials and the inclusion complexes, with a Y-offset of one unit. (**A**) Heat flow and (**B**) derivative heat flow by T, respectively.

The results are shown in Figure 8A,B. The pinostilbene profile shows two endothermic peaks at 98 °C and 118 °C (green arrow). The first peak is classically attributed to the desolvation of organic compounds, while the peak at 118 °C is the melting point of the molecule (confirmed by the manufacturer’s datasheet). The HP-β-CD also showed a large desolvation (blue arrow) at 80–100 °C [33]. As expected, the successful formation of the inclusion complex eliminated both endothermic peaks of pinostilbene, creating a smoother profile than the physical mixture (Figure 8), which still partially retains one of the two peaks. These data confirm the successful formation of the inclusion complex analyzed previously, mainly producing an encapsulation with a host/guest ratio of 1:1, despite having used HP-β-CD in excess for the formation of these complexes.

### 3.8. SEM Analysis

**Figure 9:**
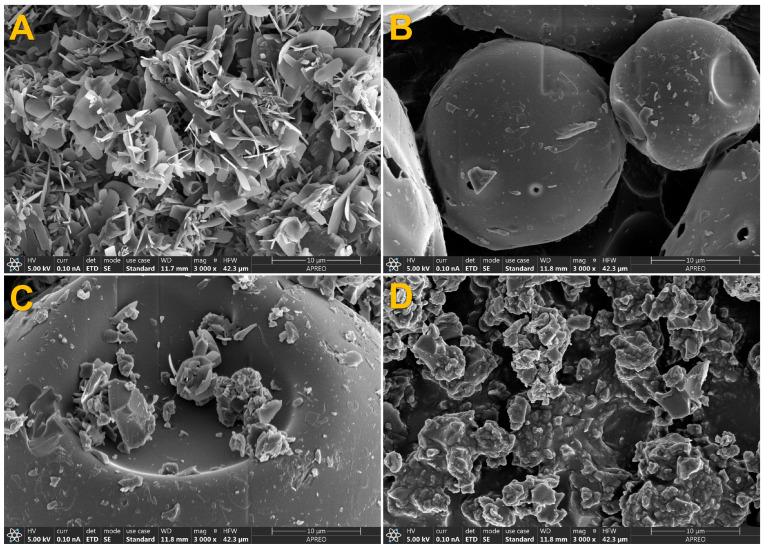
Scanning electron microscopy of (**A**) pinostilbene, (**B**) HP-β-CD, (**C**) physical mixture of pinostilbene and HP-β-CD and (**D**) pinostilbene–HP-β-CD inclusion complex.

Scanning electron microscopy shows the morphological changes that pinostilbene and CDs undergo when they are alone, physically mixed or forming inclusion complexes (Figure 9). Pinostilbene (A) forms crystals with the appearance of small, regular sheets, and HP-β-CD shows a globular structure (B). In the case of the physical mixture (C), a combination of pinostilbene and CD, with their regular structures, can be observed without morphological integration, suggesting a lack of interaction. In contrast, the images obtained from the inclusion complexes (D) show that the pinostilbene must have been included in the HP-β-CD cavity due to the morphological changes it has caused, going from a regular shape to an irregular one. These data agree with other previously published data on stilbene inclusion complexes in HP-β-CD, where the same morphological change was observed after the formation of the inclusion complexes [45].

### 3.9. Releasing-Profile Comparison

To better understand the release behavior for a potential future application, 1 mg of either free pinostilbene or pinostilbene-loaded inclusion complexes was dispersed within a 1 kDa cut-off membrane at physiological pH and temperature. In addition, it is well known that cancer cells have a more acidic environment, so an experiment at slightly acidic pH was also performed.

**Figure 10:**
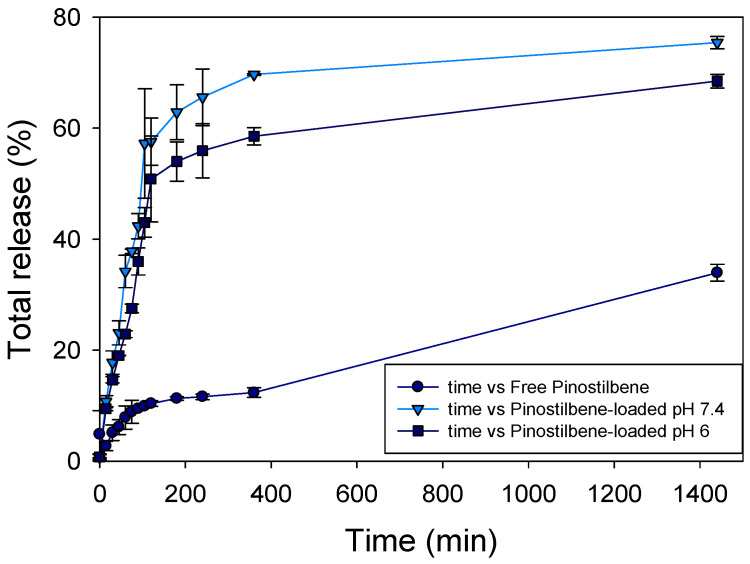
In vitro release and diffusion profile of free pinostilbene (●, diffusion) or inclusion complex (release/diffusion) using PBS at pH 7.4 (▼) or pH 6 (■) at 37 °C.

This membrane acted as the donor phase, while the receptor phase contained the same solution in a larger volume (50 mL). When comparing the CD complexes, the results (Figure 10) showed that the dissolution and subsequent diffusion of free pinostilbene across the dialysis membrane reached approximately 34% after 24 h, indicating a slower release compared to the pinostilbene-loaded formulations.

Considering only the CD-based formulations, there was a clear relationship between the pH of the medium and the release rate, since the total amount of pinostilbene remained constant across all samples.

This behavior is likely due to the amorphous nature of the drug when released from the inclusion complex, which improves solubility and enhances release [33,46]. Interestingly, all pinostilbene-loaded formulations showed higher release after 24 h, with particular attention to pinostilbene-loaded formulations at pH 7.4.

The application of the zero-order kinetic equation allowed the release constant and the regression coefficient value to be calculated. The data are shown in Table 2.

The permeability constant (K) is lower in the case of free pinostilbene, which may be due to the lower solubility of stilbene. In contrast, the presence of cyclodextrins promotes the solubility of pinostilbene and makes it more permeable to the membrane. The higher value of the permeability constant at pH 7.4 than at pH 6 can also be related to the greater solubility of pinostilbene.

Indeed, although both samples were close for 3 h, the release at 24 h was 75% at pH 7.4 in comparison with the 68% at pH 6. A possible explanation might be the higher proportion of the deprotonated form of the molecule at pH 7.4, because it is closer to its pK_a_ than at pH 6 [3].

## 4. Conclusions

In this work, the study of the antiproliferative effect of pinostilbene in colorectal cancer cells, Caco-2, has been performed; this stilbene shows similar activity to its analogue resveratrol. In order to develop drugs, cosmetics or functional foods or increase the solubility and maintain the stability of pinostilbene, a study of its encapsulation in CDs has also been conducted. The inclusion complexes formed showed a 1:1 stoichiometry; in other words, one molecule of pinostilbene is stabilized inside each CD. The CD with the highest affinity for this stilbene is HP-β-CD, which presents an encapsulation constant K_F_ = 10,074.45 ± 503.72 M^−1^ and a docking score of −10.1. Moreover, molecular docking revealed that the stabilization of the pinostilbene inside this CD takes place due to the formation of a hydrogen bond. Furthermore, the encapsulation constant is dependent on the pH of the medium: as the pH increases, the K_F_ decreases, with a sharp drop when the pK_a_ zone of the stilbene is exceeded (pH > 9). DSC and SEM results confirm that the pinostilbene complex with HP-β-CD is viable and stable, and controlled-release experiments demonstrate that pinostilbene release is enhanced when formulated within HP-β-CD and influenced by the pH of the medium. Also, the use of 1 mM HP-β-CD can triple the solubility of pinostilbene and reduce its degradation at room temperature from 31% to no more than 15% after 12 weeks of storage. All these results support the use of pinostilbene for the development of new compounds of interest to the pharmaceutical industry.

## Figures and Tables

**Table 1:** Encapsulation constants and correlation coefficients obtained by the Benesi–Hildebrand method (Equation (4)) of the inclusion complexes of pinostilbene with different CDs (25 °C, pH 7).

CD	K_F_ (M^−1^)	R^2^	
		1:1 Model	1:2 Model
α-CD	633.62 ± 31.68	0.972	0.821
β-CD	3503.62 ± 175.18	0.949	0.734
γ-CD	-	-	-
HP-β-CD	10,074.45 ± 503.72	0.976	0.868
M-β-CD	4870.24 ± 243.51	0.955	0.782

**Table 2:** Release constant and regression coefficient for a zero-order kinetic release.

Conditions	K	R^2^
Free pinostilbene	0.581	0.891
Pinostilbene—loaded pH 7.4	4.546	0.984
Pinostilbene—loaded pH 6	3.716	0.986

## Data Availability

The original contributions presented in this study are included in the article. Further inquiries can be directed to the corresponding authors.
